# Supplementary material for: Are carbon emissions trading and green financial instruments synergistic? -Comprehensive quantitative research based on content analysis
Source: PLoS One. 2024 Mar 7;19(3):e0298601. doi: 10.1371/journal.pone.0298601 (PMC10919601; doi:10.1371/journal.pone.0298601)
Supplement: S1 Data — (ZIP) [file pone.0298601.s001.zip › Data/地级市Z_F工作报告.zip!/╣π╬≈/╙±┴╓╩╨/└┤╫╘╣1⁄2╓┌║┼í╛┬φ┐╦╩2╛▌═°í┐_2018.docx]

各位代表：

现在，我代表市人民政府向大会作政府工作报告，请予审议，并请市政协委员和列席会议的同志提出意见。

一、2017年工作回顾

刚刚过去的2017年，是发展形势异常复杂严峻的一年。面对年初投资增速严重下滑、企业生产经营困难、全市经济低位运行的不利局面，在自治区党委、政府以及市委的坚强领导下，我们牢牢把握稳中求进工作总基调，牢固树立和践行新发展理念，以供给侧结构性改革为主线，以提高发展质量和效益为中心，以开展“三个年一活动”为抓手，迎难而上，主动作为，精准施策，奋勇拼搏，大力推进“五大战略”“四大攻坚”，扎实做好稳增长、促改革、调结构、惠民生、防风险各项工作，全市经济实现了低开高走、逆势上扬，各项事业持续健康发展，圆满完成了市五届人大二次会议确定的各项目标任务，在实现“两个建成”的征程中又迈出了坚实的一步。

——综合实力持续增强。预计实现地区生产总值1690亿元，增长7.5%；财政收入160.2亿元，增长5.9%；固定资产投资1672亿元，增长14%；规模以上工业增加值518亿元，增长8.5%；社会消费品零售总额726 亿元，增长10%。县域经济加快发展，北流市、容县、陆川县获评为“广西科学发展先进县”，玉州区获评为“广西科学发展先进城区”，是全区获表彰最多的市。

——项目建设持续发力。新开工重大项目310 个，竣工121个。“修机场、建码头、建高铁”三件大事有力推进，玉林民用机场全面开工建设，铁山港东岸码头建设进展顺利，玉林往广州通动车、陆川县通动车工作稳步推进。中金热轧、银亿4万吨硫酸镍扩能改造、克拉湾水上乐园等重大项目建成投产。装配式建筑与现代绿色建材产业基地建设取得重大进展，获认定为第一批国家级装配式建筑示范城市。

——改革开放持续深化。农村综合改革4项试验成果实现国家层面的政策转化。玉林高新区获批为自治区级高新区。自治区批复设立龙港新区和玉港合作园。成功承办第十七届全国村长论坛、第七届广西发明创造成果展览交易会，成功举办第十二届玉博会、第九届药博会、第七届陶博会，获评为2017年度全国优秀会展城市。

——城乡面貌持续提升。成功创建自治区文明城市，北流市成功创建国家卫生城市。海绵城市、智慧城市建设有效推进。火车站站前广场建成投入使用，风貌改造、万花楼、城区道路等城市基础设施建设加快推进。“美丽玉林·宜居乡村”建设深入开展，城乡环境综合治理扎实有效，农村人居环境不断优化。

——民生福祉持续改善。居民人均可支配收入增长9%。财政民生支出占一般公共预算支出达81.2%，增长12.5%。物价总水平保持稳定，CPI涨幅控制在2%左右。社保、健康、教育、水利、安居、农补、生态、文化、扶贫、交通等十件为民办实事工程全面完成，基本公共服务均等化程度不断提高，人民群众获得感和幸福感明显增强。

一年来，我们主要抓了以下工作：

（一）着力抓项目，在扩大有效投资中积蓄发展后劲。针对年初固定资产投资负增长的严峻形势，深入组织开展重大项目百日攻坚活动，出台征地拆迁“十四条措施”，强力推进清理整治违法用地违法建设“风暴”行动，推行项目“化零为整”“容缺后补”九项机制，落实市领导联系重大项目责任制，实行重大项目“红黑榜”通报制度，全力以赴推项目稳投资。全市固定资产投资增速迅速扭负为正，逐月稳步回升。89个自治区统筹重大项目和804个市统筹重大项目稳步推进，完成投资574.7亿元。自治区统筹重大项目开工率、投资完成率和中央预算内投资项目进度均居全区前列。在全区率先启动镇镇通二级公路工程。玉林至湛江高速公路建设步伐加快。郁江引水工程正式通水。中建科技、玉林国际汽车城等项目稳步推进，玉林（福绵）节能环保服装产业园一期建成投产，21家入园企业实现竣工，35家企业开工建设。

（二）着力抓产业，在加快产业升级中培育新供给新动能。深入实施大产业战略，着力构建"4+4+2" 产业体系。大力支持玉柴“二次创业”，稳步推进广西先进装备制造城建设，玉柴新能源特种汽车项目实现开工。加快北流家具产业园、容县生态板材家具产业园建设，北流家具大街实现开街。新开工50 个重大工业项目，竣工50个，新增规上工业企业25 家、亿元企业10家、上市（挂牌）企业2家，发展总部企业5家，十大产业产值增速均超过18%。深入实施大商贸战略，加快中国—东盟小商品交易中心、金城广场、奥园广场、万达广场等重大商贸物流项目建设，举办“岭南都会消费购物节”等品牌展会和促销活动，新增限上企业50家。开展“旅游文化发展年”活动，加快推进五彩田园、大容山、福达文化旅游体验园、岭南客家（陆川）温泉文化旅游项目等28个重大旅游项目建设，新创国家4A级景区4个。五彩花园和通往大容山景区道路建设顺利推进，实现元旦路通，春节前可实现花开。大力发展金融服务业，银行业金融机构新增贷款190亿元。深化农业供给侧结构性改革，新增广西现代特色农业(核心)示范区7 个，新增“三品一标”产品35个，容县（沙田柚）、博白县（竹木草制品）获批为国家级出口食品农产品质量安全示范区。

（三）着力抓城建，在加强建设管理中提升城市功能品质。深入实施大城市发展战略，开展“城市建设管理年”活动，推进大城市建设“一年补短板、两年立框架、三年大变样”行动计划。围绕“一核、两江、三山、四湖、五园、六组团”的大城市新格局，推进城市总体规划修编和城市规划“多规合一”。推进重大市政道路、城市新区开发、市政设施建设等十大城市建设工程，新开工城建项目278个，清宁路至城站路改造工程、一环路立交桥重建工程等一批重大项目进展顺利。积极实施玉北同城化项目，北流甘村至新圩公路改扩建工程全线通车。深入开展“四车”“泥头车”“五乱”整治，查扣违法车辆1800多辆，城市市容市貌不断改善。推行城市道路和公共场所停车收费，城市交通秩序进一步规范。深化城市管理执法体制改革，城市管理执法不断向基层延伸。全面启动全国文明城市、国家卫生城市、全区宜居城市“三城同创”工作。

（四）着力抓改革，在深化改革创新中释放发展活力。实施创新驱动发展战略，加快建设创新型玉林，科技发明专利申请量、授权量和拥有量居全区前列。深化供给侧结构性改革，落实降成本措施，继续清理涉企收费，减轻企业负担29亿元。组建广西玉林金融投资集团，成立广西第一家市级政策性农业信贷担保公司。设立政府投资引导基金、贷款周转金、贷款风险补偿金，改进“惠企贷”服务，缓解中小企业融资难题。国资国企改革不断深化，启动玉柴集团混合所有制改革。国有林场改革基本完成。深化商事制度改革，新增市场主体4.6万户。深化“放管服”改革，取消、调整行政审批事项316项，清理规范中介服务事项104项。完成市、县两级政府部门权责清单“两单融合”公布工作，规范优化市、县、乡三级行政权力运行流程。

（五）着力抓开放，在扩大交流合作中提升开放型经济水平。主动参与共建龙港新区，玉港合作园建设前期工作稳步开展，龙潭产业园“一港一路一航道”建设扎实推进，龙腾路玉林段建成通车，龙潭进口再生资源加工利用园区通过国家验收，成为国内首个口岸调往内地转检的再生资源园区。积极推进玉林市“一带一路”现代化机械装备制造业生产基地和出口基地建设，北流仲礼陶瓷马中关丹产业园项目进入国家项目库。全力推进全国通关一体化进程，落实通关时间压缩三分之一以上。深入实施第二轮“加工贸易倍增计划”，减免加工贸易企业风险担保金2.3 亿元，外贸进出口增长19.9%。继续实施招商引资三年行动计划，招商引资新落地项目251个。

（六）着力抓生态，在促进绿色发展中优化城乡环境。坚决整改中央环保督察和中央巡视“回头看”突出问题，

全面排查整治环境安全隐患，严厉打击环境违法行为。落实“水十条”“大气十条”“土十条”，全力打好污染防治“三大战役”，城乡环境持续改善。全面落实“河长制”，加大九洲江、南流江流域环境综合治理力度，九洲江水质持续稳定达到三类水标准。完成北流日用陶瓷工业园、龙潭产业园、玉林（福绵）节能环保服装产业园3家工业污水处理厂建设。加快博世科生态循环养殖产业园建设，大力推广“高架网床+益生菌”等生态养殖模式，陆川县、容县被列为国家畜牧业绿色发展示范县。大力推进宜居乡村建设，“产业富民”“服务惠民”“基础便民”专项活动全面推进，农村改厕、改厨、改圈取得阶段性成果。

（七）着力抓民生，在保障和改善民生中提升群众获得感。打好精准脱贫攻坚战，落实脱贫攻坚资金28.6亿元，发放扶贫小额贷款17.5亿元，预计实现12万多人脱贫。全力打造100个产业扶贫示范基地，新增参与扶贫的新型经营主体590家。完成易地扶贫搬迁项目20个，搬迁1.1万人，综合进度排全区第二名。发放困难群众基本生活救助资金7.1亿元。保障性安居工程建设稳步推进，开工建设棚改房7247套。城镇新增就业4.7万人，城镇登记失业率保持较低水平。投入教育事业资金93亿元，新建学校36所，改扩建学校360所，新增学位1万多个，有效缓解“入学难”“大班额”问题。县域义务教育均衡发展，陆川县、兴业县通过自治区督导评估，玉州区、容县通过国家认定。高考一本上线人数继续位居广西前列。广西医科大学玉林校区加快建设，玉林师范学院建设地方应用型高水平大学稳步推进。医药卫生体制改革不断深化，公立医院改革取得阶段性成果，医疗保障和服务水平有新提高。全市基本医疗保险参保率稳定在98%以上。公共文化服务水平不断提高，社会主义文化繁荣发展。全民健身活动蓬勃开展。

（八）着力抓治理，在创新治理体系中维护社会和谐稳定。深化“一大六小”多元矛盾纠纷调解机制，深入开展精准排查、精细化解信访积案攻坚活动，有效化解一批信访积案。全面推进网格化管理服务，推进“三官一律”进村入社，开展法律援助促进矛盾纠纷化解，信访总量下降27%。推进“天网+地网”工程建设，加快构建立体化社会治安防控体系，严厉打击“盗抢骗”“黄赌毒”违法犯罪，刑事案件总量下降15%。开展国家安全教育，筑牢国家安全人民防线。落实安全生产责任制，深入开展安全生产大检查大整治，严把安全生产关。深化火灾防控，消防安全持续平稳。落实食品安全党政同责、一岗双责，切实保障“舌尖上的安全”。群众安全感和满意度持续提升，和谐稳定局面进一步巩固。

同时，防灾减灾救灾、气象服务、防震应急、人防建设得到加强，妇女儿童、老年人和残疾人权益得到有效保障，社会救助制度日趋完善，宗教、侨务和港澳台事务取得新成效，民族、统计、供销、水文、无线电管理、方志、档案、机构编制、住房公积金管理、科普等工作取得新成绩，双拥共建、军民融合工作迈出新步伐，各项社会事业取得新进步。

一年来，我们深入开展“两学一做”学习教育，切实加强政府自身建设。深入学习贯彻落实党的十九大精神，坚决维护以习近平同志为核心的党中央权威和集中统一领导，确保中央、自治区和市委政令畅通、令行禁止。认真执行市人大及其常委会的决议、决定，自觉接受人大、政协和社会监督，认真听取各民主党派、工商联、无党派人士和人民团体的意见，人大代表建议和政协委员提案全部办结。认真贯彻落实全面从严治党要求，锲而不舍落实中央八项规定精神，促进政府系统廉洁行政。全面推进依法行政，落实司法建议，积极落实行政机关负责人出庭应诉制度，健全重大行政决策机制。加强重点领域的审计监督和行政监察。不断深化政务公开和信息公开。全面加强政务服务体系建设，机关作风进一步好转。

各位代表，过去一年的成绩来之不易。这是自治区党委、政府和市委坚强领导的结果，是市人大依法监督的结果，是市政协和社会各界监督支持的结果，是全市人民团结奋斗的结果。在此，我代表市人民政府，向全市人民，向人大代表、政协委员，向中央及自治区直属驻玉单位、驻玉部队、武警官兵，向各民主党派、工商联、无党派人士、各人民团体和海内外所有关心、支持玉林发展的各界人士，表示崇高的敬意和衷心的感谢！

在肯定成绩的同时，我们也清醒地看到，困难和问题不容忽视。经济企稳向好基础不够牢固，经济下行压力依然较大；发展不平衡不充分问题仍然比较突出，发展质量和效益还不高；土地、资金、环境等要素制约突出，重大项目投资后劲不足；改革开放广度深度仍然不够，制约科学发展的因素还比较多；一些社会事业和公共服务还比较滞后，保障和改善民生任务艰巨，等等。对这些问题，我们一定高度重视，采取有力措施，切实加以解决。

二、2018年主要工作任务

2018年是贯彻落实党的十九大精神的开局之年，是改革开放40周年，是自治区成立60周年，也是决胜全面建成小康社会、实施“十三五”规划承上启下的关键一年。党的十九大作出了中国特色社会主义进入新时代的重大政治论断，科学标定了我国发展新的历史方位和时代坐标，开启了坚持和发展中国特色社会主义的新纪元。自治区党委提出持续营造“三大生态”、加快实现“两个建成”，谱写建党百年广西发展新篇章的宏伟目标，为我们带来了重大发展机遇。我市经济平稳发展，产业持续优化，发展方式加快转变，基础设施不断完善，发展新动能日趋形成。我们要科学把握、主动担当新时代赋予的新使命，奋力谱写新时代玉林发展新篇章。

今年经济社会发展的总体要求是：以习近平新时代中国特色社会主义思想为指导，深入学习贯彻党的十九大精神和习近平总书记视察广西时提出的“五个扎实”要求，按照中央、自治区和市委的部署，坚持稳中求进工作总基调，落实新发展理念，紧扣发展不平衡不充分的问题，按照高质量发展要求，统筹推进“五位一体”总体布局和协调推进“四个全面”战略布局，深入推进“五大战略”“四大攻坚”，坚持以供给侧结构性改革为主线，以开展“三个一百三攻坚”为主抓手，统筹推进稳增长、促改革、调结构、惠民生、防风险各项工作，着力加快向海发展，着力加快产业转型升级，着力加快大城市建设，着力深化改革开放，着力保障改善民生，推动质量变革、效率变革、动力变革，建设现代化经济体系，促进经济社会持续健康发展，为加快实现“两个建成”、谱写新时代玉林发展新篇章奠定坚实基础。

经济社会发展主要预期目标是：地区生产总值增长7.5%左右；财政收入增长6%；固定资产投资增长12%；规上工业增加值增长8%；社会消费品零售总额增长10%；外贸进出口总额增长6%；居民人均可支配收入增长9%；节能减排降碳和能源消耗总量控制在自治区下达目标内；居民消费价格涨幅控制在3%左右；城镇登记失业率控制在4%以内。

新时代、新气象、新作为。实现以上要求和目标，需要我们增强使命感、责任感、紧迫感，勇做新时代的坚定者、奋进者、搏击者，以时不我待、只争朝夕的精神，解放思想、凝心聚力、敢于担当、苦干实干，积极投身玉林发展新实践，不断开创各项工作新局面。重点抓好以下工作：

（一）坚定不移融入“一带一路”，构建向海发展新格局。加快“东靠南下、通江达海”交通网络建设，打通出海通道，积极共建龙港新区，推动玉林发展由内陆开放型向临海开放型转变、由腹地经济向临海经济转变、由内陆城市向临海城市转变。

深入实施大交通战略。全力推进“修机场、建码头、建高铁”三件大事。加快玉林民用机场建设，年内完成主体工程。全力配合推进铁山港东岸码头“一港一路一航道”建设，打通玉林出海口。实现玉林往广州方向开行动车、陆川县通动车目标。积极争取张家界—桂林—玉林—海口高速铁路纳入国家中长期发展规划。加快南宁—横县—玉林—梧州城际铁路前期工作，争取年内开工建设。加快玉林至湛江、荔浦至玉林、松旺至铁山港东岸高速公路建设，开工建设浦北至北流（清湾）高速公路。加快推进绣江复航工程前期工作。完善内连外通、互联互通的综合交通基础设施，打造通江达海、陆海空联动的现代化立体交通网络。

积极共建龙港新区。加快玉港合作园规划建设，全面启动园区一期5平方公里的基础设施建设，深入开展园区产业招商活动，力争引进和开工建设一批产业项目。加快龙潭产业园开发建设，进一步完善园区基础设施和公共服务设施，扶持壮大中金、银亿、桂瑾轩、汉龙等重点企业，加快中金60万吨不锈钢薄板、银亿100万吨红土镍矿绿色开发等一批重大项目建设，拓展延伸不锈钢、合金等产业链，做强做大有色金属产业，培育壮大新材料、再生资源、临港产业三大主导产业。

打造向海发展平台。加快再生资源固体废物进口口岸建设，完善海关、检验检疫监管场所配套设施，实现龙潭进口再生资源加工利用园区封关运营。积极推进保税物流体系建设。规划建设玉林跨境电子商务港，完善跨境电商平台。推进关检“三个一”改革、“单一窗口”建设和全国通关一体化，构建高效便捷的大通关平台。

（二）坚定不移抓项目扩投资，掀起项目建设新高潮。围绕产业布局和城乡发展，深度谋划、强力推进一批项目建设， 全面夯实经济社会发展根基。

强力推进项目建设。加快推进9个自治区百项重大工业项目、51个重点推进工业项目、16个自治区成立60周年大庆工程项目。扎实推进自治区和市层面统筹项目，以及债券项目、中央预算投资项目建设。开工建设北流市新能源电池、福绵六万山风电等重大项目，实现玉林坡塘至民用机场公路、博白马子岭风电、红星美凯龙家居商城等重大项目竣工使用。加快龙云灌区工程以及秦镜、蕉林、黄章水库等水利项目前期工作，争取年内开工建设。

加强项目谋划储备。聚焦十大产业、基础设施、公共服务等领域，深入研究和谋划一批夯基础、补短板、增后劲的大项目、好项目。积极策划推出一批经营性项目和公共基础设施项目，发布一批PPP合作项目。紧跟中央、自治区的政策投资导向，在“一带一路”、县域经济、乡村振兴、先进制造业、互联网、大数据、旅游文化、脱贫攻坚等领域谋划储备一批重大项目、重大工程，争取更多项目列入国家和自治区盘子。

突破项目瓶颈制约。全面盘活存量土地，清理处置闲置土地，强力推进土地整治、旱改水、土地开垦复垦等工作，破解耕地占补瓶颈难题。高效推进征拆、打违、处遗、安置“四位一体”工作，深入开展“两违”整治“风暴”行动。积极拓宽融资渠道，完善信贷融资、债务置换、债券发行、PPP、基金等融资方式。发展壮大投融资实体、融资担保机构，创新投融资机制，增强融资能力。着力推动民间资本投向实体经济。落实扩大民间投资政策措施，力争完成民间投资1400亿元。

（三）坚定不移实施大产业战略，培植产业发展新体系。大力优化产业结构，培育新动能新供给，促进产业集聚发展，推动产业迈向中高端。

实施大产业百项工程。计划推进产业项目142个，实现开工58个、竣工44个，完成投资196.4亿元。加快广西先进装备制造城和现代农机装备产业园、玉柴新能源特种汽车、玉林（福绵）节能环保服装产业园（二期）、中滔博白节能环保产业园、装配式建筑与现代绿色建材产业基地等项目建设，促进产业做强做大。创新园区管理机制，推行园区市场化、公司化管理体制和运行模式，鼓励引导行业资金和社会资金参与园区建设。优化升级机械制造、服装皮革、健康食品、新型建材、陶瓷、林产林化等传统优势产业，加快发展新材料新能源、节能环保再生资源、医药制造、新一代电子信息等战略性新兴产业，打造“4+4+2”产业新体系。

扶持企业发展壮大。扩大中小企业发展产业基金效应，采取“政银企保”一体化扶持模式，整合政府和社会资源，支持企业壮规模、提实力，力争新增规上工业企业30家、亿元以上工业企业15家。深入实施创新驱动发展战略，力争引进转移科技成果产业项目20项以上，新增高新技术企业8家。加快玉林高新区建设，实现玉林市科技企业孵化大楼建成运营，入孵企业达30家以上。加快国家陶瓷重点实验室等研发平台建设。激发和保护企业家精神，鼓励社会更多主体投身创新创业，支持民营企业发展。大力弘扬劳模精神、工匠精神。深入推进质量强玉战略，加快品牌培育，打造玉林制造品牌。

实施商贸文旅百项工程。计划推进商贸文旅项目81个，实现开工35个、竣工10个，完成投资112.5亿元。加快现代服务业集聚区建设，继续推进金城广场、奥园广场、万达广场等项目。做强一批专业市场。大力发展金融、现代物流、科技服务、商务服务、文化创意、现代商贸、健康养老等服务业。加快培育电子商务、数字经济、创意经济等新业态新模式。加快36个重大旅游项目建设，推进“五彩田园”、大容山创建国家5A级景区，加快六万山风景区创建自治区级生态旅游示范区、自治区旅游度假区，打造一批旅游品牌，大力创建国家健康旅游示范市。积极推进容县创建国家全域旅游示范区、北流创建广西特色旅游名县。

（四）坚定不移实施大城市战略，实现城市建设新突破。深入推进城市建设“一年补短板、两年立框架、三年大变样”行动计划，提升城市承载力和综合竞争力，加快建设田园休闲、生态宜居、组团式融合发展的区域性大城市。

完善城市总体规划。坚持“全域玉林”理念，推进多规合一、多规融合，加快城市总体规划修编，完善城市专项规划和玉北同城化规划，努力构建“一核、两江、三山、四湖、五园、六组团”城市发展格局，拉开大城市框架，拓宽城市发展空间，增强城市辐射带动能力。大力推进建设用地控制性规划全覆盖，争创全国新一轮土地利用总体规划编制试点城市。开展龙云灌区、南流江、清湾江上游区域水资源配置规划研究。

实施大城市百项工程。计划推进大城市项目118个，开工97个、竣工37个，完成投资139.1亿元。加快城市新区开发，规划建设高铁新城、城北中央商务区、玉东文体路生态城等城市片区开发项目，大力发展城市经济、楼宇经济。开工建设杭州绿城、上海绿地等知名企业开发的高品质居住小区。统筹推进城市道路、防洪排涝、交通治堵、公园绿地、环卫设施等市政项目建设，规划建设环城高速公路东段，改扩建二环南路，接通二环西路，实现二环路全线通车；打通新民路、金鞍路、江南大道延长线、中山路等9条道路；对苗园路、大北路、江滨路、教育中路、民主中路等5条主干道进行“白改黑”；对教育东路、教育中路、一环东路等3条道路进行风貌改造；完成15条小街小巷改造；完成火车站片区、江南片区排涝工程建设；新建、扩建东岳市场、南桥市场、镇忠市场、绿杨市场等10个农贸市场，新建4处小型垃圾转运站、3个公共立体停车场、40座公厕。稳步推进地下空间开发，统筹推进地下综合管廊建设，完善城市配电网、通信网等公共设施。加快智慧城市、海绵城市建设。启动福绵区10平方公里新区规划建设。加快玉北同城化步伐，抓好郁林大道、发展大道、玉北大道等4条主要通道建设，推进同城化制度改革，促进交通体系、城镇规划、产业发展、生态建设和公共服务一体化发展。

提高城市管理水平。深入开展全国文明城市、国家卫生城市、全区宜居城市“三城同创”工作，促进城市内涵式发展。开展城市交通秩序、市容环境、市场秩序、房屋租赁专项整治，严格落实《城市市容和环境卫生管理条例》。大力推进街区制，加快城市社区建设，完善数字化城市管理平台，实现精细化、网格化、数字化管理。深化城市管理体制改革，创新城市治理方式，推动城市治理重心下移。

（五）坚定不移壮大县域经济，培育经济发展新优势。发挥县域特色，推动县域经济提质增效、整体进位，形成县域竞相发展、充分发展的良好格局。

大力推动县域差异化发展。严格按照主体功能区定位，分类推进北流、福绵、玉东新区重点开发区，容县、陆川、兴业、博白农产品主产区和玉州城市主城区发展。建立县域经济发展激励机制，制定支持县域经济加快发展政策措施，激发县（市、区）发展活力。大力开展县域经济发展三年行动计划，推进县域基础设施、公共服务设施和产业发展三个专项计划，实施基础设施项目93个，总投资140亿元，推进23个镇镇通二级公路项目，夯实县域发展基础，做大做强县域经济，争创自治区科学发展先进县（城区）、进步县（城区）。

大力发展县域支柱产业。按照不同县域资源禀赋、区位特点、产业基础，发展县域特色产业，各县（市、区）集中力量培育发展2～3个主导产业，构建独具特色的县域产业体系。加快县域产业园区规划建设，推进北流家具产业园、容县生态板材家具产业园、博白“两江”产业转移园、玉林市新材料生态产业园等园区发展，提升县域产业园区承载能力。

大力推进县域城镇化。深入实施大县城战略，统筹抓好县城基础设施建设、产业发展、功能完善和品位提升等工作，把县城做大、做强、做优、做美。加快列入自治区“百镇扶持壮大工程”重点镇建设。各县（市、区）规划建设1～2个生态宜居示范镇。推进玉东健康小镇、北流陶瓷小镇、玉州花卉小镇、容县柚子小镇、陆川铁锅小镇、博白那林鱼小镇、兴业运动小镇、福绵服装小镇等建设，打造一批特色小镇，增强县域发展新动能，促进农民就地城镇化，构建中小城市和小城镇协调发展新格局。

（六）坚定不移实施乡村振兴战略，推进“三农”工作新发展。坚持农业农村优先发展，建立健全城乡融合发展体制机制和政策体系，推动农业全面升级、农村全面进步、农民全面发展。

推进农业农村现代化。制定乡村振兴战略实施规划，构建现代化农业产业体系、生产体系、经营体系。稳定农村基本经营制度，鼓励采取专业合作、股份合作、土地流转、土地托管等多种形式，发展农业适度规模经营。积极培育新型农业经营主体，新发展50个农民专业合作社示范社、20家示范性家庭农场。加快推进农业机械化，提升水稻生产全程机械化水平。落实强农惠农政策，支持新型农业经营主体发展农产品加工，提升农产品加工设施装备水平。通过“政银担”合作，撬动更多资金投入农业发展。开展新型主体带头人轮训计划，培养大批新型职业农民。

发展特色农业产业。深化农业供给侧结构性改革，推动农业从增产转向提质。严格落实耕地保护制度，抓好高标准农田建设，稳定粮食生产。大力发展水果、中药材、畜禽、水产、食用菌、马铃薯、蔬菜等优质特色农产品及精深加工。实施现代特色农业示范区建设增点扩面提质升级三年行动，建成自治区核心示范区4个、县级示范区14个、镇级示范园40个和一批村级示范点。大力培育农产品品牌和生态原产地保护产品，积极争创福绵（八角）国家级出口食品农产品质量安全示范区，抓好陆川橘红、北流百香果、玉林八角等农产品申报地理标志证明商标，支持申报国家生态原产地产品保护示范区。

促进农村三产融合发展。大力实施“互联网+农业”，发展特色种养业、农产品加工业、农村服务业，促进农村一二三产业融合发展。培育和引进一批农产品加工龙头企业，加快中国—东盟农产品深加工产业园、桂牛乳业10万吨乳制品等重点项目建设，打造一批三产融合示范区。加快推进五彩田园种业小镇和农业部植物新品种测试玉林分中心项目前期工作。大力发展生态休闲农业与乡村旅游，打造一批乡村旅游精品线路。

（七）坚定不移深化重点领域改革，激发科学发展新动力。坚持把改革作为推动发展的强大动力，积极深化重点领域和关键环节改革，不断释放改革红利。

深化各领域改革。打好供给侧结构性改革攻坚战，推进“三去一降一补”，进一步优化产业结构。深化财税改革，建立权责清晰、财力协调、区域均衡的市与城区财政关系。加强政府性债务风险评估、预警和监管，防范财政风险。深化金融体制改革，组建地方政府金融监督管理机构，打好防范化解金融风险攻坚战。加快不动产信息平台建设，完善不动产登记制度。继续推进农村信用合作联社改制为农村商业银行。推进经营性事业单位改革。深化医药卫生体制改革和公立医院改革，加快医疗联合体建设。加快住房制度改革和长效机制建设，加快建立多主体供应、多渠道保障、租购并举的住房制度。不断完善住房公积金管理制度。深化投融资、价格、供销社、科技、文化、生态文明等领域改革，激发市场活力和社会创造力。

深化农村综合改革。抓好全国农村改革试验区、农村集体经营性建设用地入市、农村土地征收制度改革、农村宅基地改革等国家改革试验任务。不断深化农村土地确权登记颁证及所有权、经营权、承包权“三权分置”改革，巩固扩大国有林场、集体林权制度改革成果。加快健全农村产权流转交易市场体系，完善市县镇村“四级”平台综合服务功能，促进农村产权规范有序流转。大力推进农村土地承包经营权抵押融资，加快建立全市农村土地承包经营权抵押贷款统一登记制度。深化农村集体产权制度改革，保障农民财产权益，壮大农村集体经济。

深化国资国企改革。实施国有企业违规经营投资责任追究暂行办法，规范企业经营行为。加快玉柴集团混合所有制改革，推进玉柴集团“二次创业”。加快推进政企分开，实现经营性国有资产集中统一监管，政府平台公司全部完成经营性转型。整合资源，做大做强市城投公司、玉林交旅集团；通过重大项目实施和业务拓展，做实玉林投资集团和玉东投资公司；在支持产业发展、拓展担保业务中壮大金融投资集团和农业担保公司。

持续优化营商环境。开展优化营商环境集中行动，继续加大“放管服”改革力度，着力打造稳定公平透明、可预期的营商环境。进一步落实简化审批环节、提高审批速度九条措施，继续完善行政审批“容缺后补”“圆桌”会议、“化零为整”等机制。深化商事制度改革，全面推进“多证合一、一照一码”，推进“证照分离”试点。改进监管方式，减少市县两级政府执法种类，推进跨地区、跨部门的综合执法，完善以“双随机、一公开”为主要方式的事中事后监管方式。大力支持非公经济发展，营造尊重、激励和保护企业家干事创业的社会氛围。

（八）坚定不移实施开放带动战略，拓展开放合作新空间。以更开放的姿态、更大的工作力度，深化对外开放合作，发展更高层次的开放型经济，加快形成开放合作新格局。

全面深化开放合作。加快“一带一路”现代化机械装备制造业生产基地和出口基地建设，大力推进中泰（玉林）国际旅游文化产业园、中国—文莱（玉林）健康产业园等合作项目，打造跨境产能合作发展示范基地。深化与长三角、粤港澳大湾区等地区的合作，积极推进与广东共建合作园区，加快海峡两岸农业合作试验区建设，积极对接先进生产力。力争北部湾城市同城化政策扩展到玉林，实现一体化发展。

大力推进精准招商。开展精准招商“一月一专题、每季一活动”，围绕“五大战略”“四大攻坚”，着力引进一批世界500强、中国500强、民企100强，以及上市企业、行业百强企业到玉林投资发展。发挥玉林侨乡优势，利用好玉博会、药博会、陶博会等平台，吸引在外创业的“玉商”回归。推动意向项目签约，抓好中船重工、中建集团、中仿智能、中航工业、宝能集团、柳钢集团、源盛物流、华润医药等一批项目落地发展。

积极拓展对外贸易。深入实施“引资强贸”工程，推进第二轮“加工贸易倍增计划”，鼓励拥有自主品牌和自主知识产权的企业扩大出口，促进加工贸易产业集群发展。以“一带一路”建设为引领，加快机械、陶瓷、医药、食品、服装皮革等优势产业走出去，不断增强企业市场竞争力。

（九）坚定不移推动绿色发展，建设美丽生态宜居新玉林。坚持绿色发展，加强生态文明建设，实现更高质量、更有效益、更可持续的发展，努力满足人民日益增长的优美生态环境需要。

实施生态环境治理攻坚。计划推进生态建设项目56个，实现开工13个、竣工17个，完成投资19.4亿元。加快市污水处理厂升级改造工程、玉柴工业园污水治理工程、“金山银山”工程重点区域林相改造等一批生态环保项目实施，确保主要污染物排放总量继续减少，生态环境质量总体改善。严格落实“河长制”，继续全面推进九洲江、南流江流域环境综合治理，坚决打赢“两江”污染治理攻坚战。加强重点饮用水源治理，确保群众饮水安全。加强大气污染整治，着力提高大气环境质量。改革生态环境监管体制，全面完成中央环保督察突出问题整改，坚决惩处破坏生态环境行为。

着力发展生态经济。培育壮大生态工业、生态农业和生态服务业，加快绿色园区、绿色工厂、绿色企业、绿色建筑、绿色矿山建设。加快发展节能环保产业，打造“城市矿产”再生资源循环产业基地。大力发展生态循环经济，加快实施养殖废弃物资源化利用整县推进项目，积极推进博世科生态循环养殖产业园建设，打造生态养殖、废弃物处理、产品精深加工、特色种养、商贸冷链物流的全产业链生态养殖模式。

着力优化人居环境。深入实施大田园战略，加快五彩花园、五彩果园、五彩菜园、五彩稻园、五彩药园建设，争创国家级田园综合体，打响“五彩玉林·田园都市”品牌。建设玉林—北流—容县生态景观带，推进绿色城镇化。开展大规模国土绿化行动，实施城乡绿化工程，扩大绿化空间，提升绿化品质。持续深化“美丽玉林”乡村建设，加强农村环境综合整治，抓好农村生活污水治理，进一步改善农村人居环境，大力建设国家生态文明先行示范区。

（十）坚定不移增进民生福祉，努力创造美好新生活。坚持以人民为中心的发展思想，解决突出民生问题，补齐民生短板，增进民生福祉，实现社会治理更加和谐稳定。

实施脱贫攻坚。坚持精准扶贫、精准脱贫，围绕“两不愁三保障”，推进大扶贫战略行动，全面实施“六个全覆盖六个绝不漏”攻坚行动，确保13.2万贫困人口脱贫，136个贫困村脱贫摘帽，巩固陆川县脱贫摘帽成果，实现兴业县脱贫摘帽，扎实推进博白县脱贫攻坚。计划完成村屯道路建设120条900公里、危房改造7700多户、易地扶贫搬迁5100多人。实施“三个100产业扶贫工程”，发展壮大贫困村集体经济。瞄准特定贫困群众精准帮扶，进一步向深度贫困地区聚焦发力，巩固扶贫成果，提高脱贫质量，坚决打赢精准脱贫攻坚战。

实施学校建设攻坚。计划推进学校建设项目105个，实现开工46个、竣工19个，完成投资23.1亿元。加大教育投入，确保教育经费“三个增长”，努力做到幼有所育、学有所教。落实立德育人根本任务，坚持发展素质教育。实施第三期学前教育行动计划，新建一批公办幼儿园，新增多元普惠幼儿园160所。全面推进义务教育学区制管理改革，推动县域义务教育均衡发展，力争博白县通过自治区督导评估，陆川县、兴业县通过国家认定。启动实施普及高中阶段教育攻坚计划，加快市一中新校区、福绵高中迁建工程、市十中迁建工程等项目建设。加快广西医科大学玉林校区建设，支持玉林师范学院建设高水平地方应用型大学和硕士学位授予单位。加快玉林职业教育园区和玉林职业技术学院规划建设。加大科普工作力度，提高全民科学素质。

努力扩大就业。深入实施就业优先战略和积极就业政策，推动创业带动就业。加快基层就业和社保服务中心建设，深入实施就业脱贫工程，大力推进创业孵化基地、农民工创业园等创业就业平台建设，加大创业担保贷款扶持力度。做好高校毕业生、城镇就业困难人员、农村转移劳动力等各类群体就业工作。

推动文化繁荣兴盛。挖掘、开发和保护岭南文化、玉商文化、侨乡文化、客家文化、乡贤文化等本土文化资源，打造特色文化精品。加快中国—东盟绿色创意印刷园玉林园区建设。推进市档案馆新馆、科技馆、工人文化宫、群众艺术馆新馆、王力博物馆等项目建设。加快建设万花楼、岭南文化特色街等一批文化特色街区。推进传统媒体融合转型，加快新闻、广播、电视事业发展。

推进健康玉林建设。实施全民健康素养促进行动计划、基层医疗卫生机构能力建设行动计划，开工建设玉林桂南益寿园医养结合项目，竣工市颐养园（一期）等项目。加快广西医科大学玉林校区附属医院、市骨科医院整体搬迁工程、市儿童医院等医疗机构建设，加大社区、镇村卫生服务机构建设力度，增加医疗卫生公共服务供给，努力缓解群众“看病难、看病贵”问题。完善全民健康信息服务体系，推进健康医疗大数据应用。坚持中西医并重，推动中医药事业发展。支持社会办医，发展健康服务产业。大力实施健康产业三年专项行动计划。加强食品药品安全监管，全力创建广西食品安全示范城市。加强公共体育设施建设，广泛开展全民健身运动，举办第四届玉林市运动会。

完善社会保障体系。加大社保扩面征缴力度，继续阶段性降低社保缴费费率。深入推进社会保障“一卡通”工作。继续实施城乡居民基本医保制度和大病保险制度，建立职工大额补充医疗费用统筹制度，推进异地就医住院费用直接结算工作。采取有力措施，确保农民工工资支付落到实处。完善社会救助、社会福利、慈善事业、优抚安置等制度，推进市复员退伍军人养老服务中心、复员退伍军人综合楼建设。加快养老服务体系建设，努力实现老有所养、老有所学。大力实施妇女儿童“两个规划”，促进妇女儿童事业全面发展。保障未成年人、残疾人合法权益。

继续实施为民办实事工程。全力抓好社保惠民、健康惠民、教育惠民、水利惠民、安居惠民、农补惠民、生态惠民、文化惠民、扶贫惠民、科技惠民等十大类为民办实事工程项目。

加强社会治理创新。培育和践行社会主义核心价值观，加强理想信念教育和思想道德建设。巩固提升国家公共文化服务体系示范区创建成果，深化群众性精神文明创建活动。加大全民普法力度，加快公共法律服务体系建设。完善党委领导、政府负责、社会协同、公众参与、法治保障的社会治理体制，提高社会治理社会化、法治化、智能化、专业化水平，打造共建共治共享的社会治理新格局。全面推行网格化管理，推动社会治理重心向基层下移，实现政府治理和社会调节、居民自治良性互动。加强农村基层基础工作，健全自治、法治、德治相结合的乡村治理体系。加强预防和化解社会矛盾机制建设，推进信访工作基层基础标准化、业务规范化、队伍专业化建设，积极推行网上信访。加快推进“雪亮工程”建设，完善社会治安立体防控体系，依法防范和打击“黑枪毒”“盗抢骗”等违法犯罪行为，保护人民群众的人身权、财产权和人格权，巩固提升人民群众的安全感和满意度。强化安全生产责任，加大安全隐患排查整治防范，坚决遏制重特大安全事故。加快综合应急救援基地和指挥系统建设，全面提升社会应急救援能力。加强防震减灾工作。建成市县两级突发事件预警信息发布系统，基本实现气象现代化。加强国家安全教育，落实国家安全责任。加强国防动员和后备力量建设，支持国防和军队深化改革。深化双拥共建工作，巩固“全国双拥模范城”创建成果，深入推动军民融合发展。

三、全面加强政府自身建设

各位代表，进入新时代，我们将以习近平新时代中国特色社会主义思想为指导，始终践行全心全意为人民服务的宗旨，不忘初心、牢记使命，不断提高驾驭经济、管理社会、推进改革、服务群众的能力，深入建设为民、务实、清廉的政府。

落实全面从严治党。严格落实《中共中央政治局关于加强和维护党中央集中统一领导的若干规定》精神。严格执行新形势下党内政治生活若干准则，推进“两学一做”学习教育常态化制度化，开展“不忘初心、牢记使命”主题教育。持之以恒抓好作风建设，巩固拓展落实中央八项规定精神成果，在反对形式主义、官僚主义上下更大功夫，一个节点一个节点坚守，一个问题一个问题突破，移风易俗、成风化俗。强化扶贫领域监督执纪问责，加大整治群众身边腐败问题力度。改革审计管理体制，推进审计全覆盖。

优化政府服务供给。深化“互联网+政务服务”，制定和公开实施“零跑腿”事项清单，打造24小时“不打烊”网上政府，实现审批、公共服务“零跑腿”事项分别达到50%、80%以上，其他事项最多跑一次。全面推进政务公开，推动决策、执行、管理、服务和结果“五公开”。 深化公共资源交易工作，打造“阳光型”政府交易平台。深化行政管理体制改革，加快政府职能转变，努力做到体制顺、机制活、效率高、服务好。

加强法治政府建设。强化法治思维，落实法治责任，推进依法行政。实行行政机关负责人出庭应诉制度，支持检察机关依法开展公益诉讼。严格按照法定权限、法定程序行使职权，推进重大决策评估，严格执行人大及其常委会决定，自觉接受人大依法监督和政协民主监督，认真听取人大代表、政协委员和各民主党派、工商联、无党派人士、人民团体的意见建议，提高人大代表建议和政协委员提案办理质量。主动接受社会监督和舆论监督，让人民群众更有效地监督政府。

强化真抓实干作风。坚持实干兴玉，把真抓实干作为政府工作的座右铭，突出“干”字当头，推行“马上就办”，对定下来的事情雷厉风行、抓紧实施，对部署的工作加强督查、一抓到底，以钉钉子的韧劲，克难攻坚，落到实处，确保各项工作取得实效。强化激励机制，让想干事、能干事、干成事的干部受到激励和表彰。加大行政问责力度，加强政务督查和政风监督，坚决纠正不作为、乱作为，严惩懒政怠政、失职渎职行为。

各位代表，新时代赋予新使命，新使命需要新担当！让我们更加紧密团结在以习近平同志为核心的党中央周围，坚持以习近平新时代中国特色社会主义思想为指导，全面贯彻落实党的十九大精神，在市委的坚强领导下，不忘初心、牢记使命，苦干实干、狠抓落实，为加快实现“两个建成”、全面开启现代化建设新征程、谱写新时代玉林发展新篇章而努力奋斗！
